# Opening up the black box of recovery processes in persons with complex mental health needs: a qualitative study of place-making dynamics in a low-threshold meeting place

**DOI:** 10.1186/s13033-022-00560-9

**Published:** 2022-10-14

**Authors:** Clara De Ruysscher, Stijn Vandevelde, Stijn Vanheule, Dirk Bryssinck, Wim Haeck, Wouter Vanderplasschen

**Affiliations:** 1 Department of Special Needs Education, Ghent University, Henri Dunantlaan 2, 9000 Ghent, Belgium; 2 Department of Psychoanalysis and Clinical Consulting, Ghent University, Henri Dunantlaan 2, 9000 Ghent, Belgium; 3 Villa Voortman, Vogelenzangpark 10-17, 9000 Ghent, Belgium

**Keywords:** Complex mental health needs, Recovery, Place-making dynamics, Rituals

## Abstract

**Background:**

The recovery processes of persons with complex mental health needs take a slow and unpredictable course. Despite the fact that a number of essential building blocks of recovery in this population have been identified (e.g. social relationships, treatment, personal beliefs), the actual process of recovery in persons with complex mental health needs largely remains a black box. The aim of this study was to gain insight into how the recovery processes of persons with complex mental health needs take place, by applying a relational geographical approach and scrutinizing the place-making dynamics of one low-threshold meeting place in Belgium engaging with this group.

**Methods:**

Data collection took place during the height of the COVID-19 pandemic by means of 11 in-depth interviews with different involved actors (service users, staff members, volunteers) and analyzed thematically.

**Results:**

Results showed how the daily practice of the meeting place is continuously reproduced through place-making rituals that create an inclusive space of hospitality, are fueled by creative processes and form an indispensable counterweight for service users’ mental health needs.

**Conclusions:**

To further open up the ‘black box’ of recovery in persons with complex mental health needs, it is vital to focus our analytic gaze onto recovery as a dynamic and relational practice.

## Introduction

From the early 1980s onwards, persons with co-occurring mental health and substance use disorders came into the picture of mental health care research and practice as a specific and ‘hard to reach’ population [1]. Today, despite the conflicting diversity of reported prevalence numbers, it is widely acknowledged that this comorbidity is frequent: up to 50% of persons with substance use problems also have severe mental health problems and vice versa [2–5]. As their daily lives are shaped by an accumulation of several personal and social problems (e.g. severe mental health problems, substance use, homelessness, poverty, judicial problems, a poor physical health, social isolation, stigma), the recovery processes of persons with complex mental health needs take a slow and unpredictable course and are characterized by intense ups and downs [6].

In recent years, international mental health care has made a transition towards recovery-oriented and integrated systems of support, in which recovery is predominantly described as *“a deeply personal, unique process of changing one’s attitudes, values, feelings, goals, skills and roles. It is a way of living a satisfying, hopeful, and contributing life, even with limitations caused by illness. Recovery involves the development of new meaning and purpose in one’s life as one grows beyond the catastrophic effects of mental illness”* (7, p. 527). In line with this transition, recent recovery research focusing on persons with complex mental health needs has identified a number of factors contributing to these recovery processes, such as social relationships (e.g. the role of peers, family support, community belonging), treatment-related factors (e.g. individualized care, therapeutic relationships, medication, psycho-education), sobriety or controlled substance use, meaningful activities, and personal beliefs (e.g. hope, identity, self-responsibility, self-determination, spirituality) [8–12]. These factors could be considered building blocks of recovery in persons with complex mental health needs and tally well with popular conceptualizations of personal recovery such as the CHIME-D framework, in which Connectedness, Hope, Identity, Meaning in life, Empowerment and coping with Difficulties are put forward as central recovery processes [13, 14]. However, the question as of *how* recovery processes of persons with complex mental health needs come about remains largely unanswered [8]. In other words, although we have a good understanding of the building blocks, the actual *process* of dual recovery largely remains a ‘black box’.

A possible explanation for the reason why we still struggle to open up this ‘black box’ of recovery in persons with complex mental health needs can be found in the growing body of critique that recovery has been conceptualized and studied too one-sidedly as a personal (and even individual) journey, diverting attention away from the deeply relational character of recovery [15]. From a relational stance, recovery processes such as rebuilding one’s identity, feeling hopeful about the future and empowerment are not merely facilitated or undermined by contextual factors, but are fundamentally impossible to imagine or conceive outside of these contexts [11, 15]. In the case of persons with complex mental health needs, their recovery processes cannot be disconnected from their complex everyday realities (e.g. street life, poverty, social isolation, judicial problems, previous treatment experiences, stigma), as they are the scenes in which recovery comes about [16]. Insights from the field of relational geographies, in which the focus lies on the enabling (e.g. 17), restorative (e.g. 18) or therapeutic (e.g. 19) dimensions of places and environments, help us to further deepen and concretize this train of thought on relational recovery. For example, a meta-synthesis of Doroud, Fossey & Fortune [20] identified four interconnected mechanisms through which recovery processes and place are inextricably linked: being (e.g. having a sense of security and stability), doing (e.g. engaging in meaningful activities), becoming (e.g. overcoming challenges, setting goals) and belonging (e.g. connecting with others) [20].

From a relational geographical perspective, dynamics between recovery and context are described as processes of *place-making*, in which places do not pre-exist as stable entities, but are seen as active concentrations of dynamics that are continuously (re)shaped and (re)produced in human interaction [21–25]. In that sense, place-making dynamics resonate well with Durkheim’s work on the significance of rituals, that are described as symbolic actions through which community identities are built, unique community values are reaffirmed, and collective representations and enactments of these values are produced [26]. Thinking alongside the lines of Goffman’s interpretation of Durkheim’s theory [27, 28], rituals do not exclusively serve the purpose of specific social ceremonies (e.g. commemoration), but are an essential part of everyday life, in which encounters play a pivotal and dynamic role in shaping the moral order [29, 30]. Translating this conceptualization of rituals to the context of this study, place-making dynamics could be interpreted as strings of ritualistic interactions through which recovery resources are produced.

If we want to open up the ‘black box’ of recovery and recovery-oriented practice for persons with complex mental health needs, it is crucial to reorient our focus onto the ways they experience their context and engage in place-making dynamics in search of adequate resources for recovery. Therefore, the aim of the current study is to gain understanding of how the recovery processes of persons with complex mental health needs ‘take place’ in their most literal sense. We will do this by scrutinizing the place-making dynamics and rituals of one low-threshold meeting place for persons with complex mental health needs in Ghent (Belgium), called Villa Voortman [31].

Previous research into the daily practice of this meeting place has been focusing on the functions it fulfills in the lives and recovery processes of its service users [32]. The results showed how on the one hand, the meeting place is experienced as *a place to be*, i.e. a safe place where visitors feel welcome and accepted, and gradually even start to feel ‘at home’. On the other hand, the meeting place also functions as *a place to be me*. Villa Voortman is a place bursting with creative and artistic activities (e.g. making music, painting, sculpting, dancing, poetry writing). Through these creative activities that are rooted in personal interests and talents, visitors find a language to re-engage in dialogue with others. In doing so, they start rebuilding positive identities that move away from other stigmatizing identities and become visible citizens in society. Whilst these results show that service users find material, social and affective recovery resources in Villa Voortman, the study failed to unravel the place-making dynamics and rituals through which these resources are produced.

However, to understand *how* recovery processes of persons with complex mental health needs take place, studying these underlying dynamics is of vital importance. In that sense, the global COVID-19 pandemic unexpectedly provided us with a unique research opportunity. The pandemic’s societal impact and preventive corona measures severely disturbed the meeting place’s daily practice and prevailing place-making dynamics. This created a unique caesura in which the actors of the meeting place suddenly found themselves confronted with the partial and temporary suspension of their familiar rituals, and were challenged to actively search for ways to re-establish, recreate and reinvent the dynamics through which the meeting place’s identity and recovery resources are produced. In that sense, the COVID-19 pandemic provided us with a rare magnifying glass on the place-making dynamics of the meeting place.

## Methods

### Methodological approach

This study is rooted in a relational geographical conceptualization of place. To gain understanding of the ways recovery-enabling places are created, this study takes the shape of an in-depth qualitative analysis of the place-making dynamics of one enabling place. Doing justice to the idiosyncrasy of recovery processes and the complexity of place-making dynamics, data collection was rooted in the personal perspectives and lived experiences of the meeting place’s daily users, i.e. service users (called ‘visitors’) and staff members. Data were collected by means of in-depth interviews and analyzed thematically.

### Research location and participants

As outlined in the Introduction section, the scene of this study is Villa Voortman, a low-threshold meeting place in Ghent (Belgium) targeting persons with complex mental health needs who, due to their complex mental health needs, are often confronted with social exclusion mechanisms and risk falling between the cracks of regular treatment modalities. Recognizing the precarious situation of this group and in an attempt to challenge these barriers to recovery, the meeting place aims to offer an alternative approach in which human encounters and artistic projects are placed at the heart of its practice. The daily practice is run by a diversity of staff members (e.g. mental health nurses, psychologist, creative therapist), volunteers and interns. The meeting place is open on a daily basis (except for Sundays) and is used by about 15 to 25 different service users per day, called ‘visitors’. Characteristic of Villa Voortman is that it is a homely rather than a clinical place, underpinned by a horizontal organizational structure. A wide array of voluntary activities (e.g. sports, yoga, making music, art) and so-called ‘ateliers’ (e.g. philosophy, poetry writing) are offered, organized by both visitors, staff members and volunteers. Additionally, based on the talents of the visitors, Villa Voortman is involved in the creation and performance of several large-scale music and theatre productions in the professional cultural circuit.

Data collection took place at the time of the global corona crisis, which had a profound impact on the daily routines of Villa Voortman. At the time of writing this article (April 2021), Belgium found itself in the middle of a third local wave of the COVID-19 pandemic and consequential lockdown measures. During the 1 week of the first Belgian lockdown, the meeting place had to close its doors to visitors and to reduce its service drastically. Consequently, the only function the staff of the meeting place could fulfill was to hand out take-away meals and stay in touch with visitors ‘through the window’. Gradually, Villa Voortman managed to find a more adequate balance between providing tailor-made support on the one hand and following the imposed corona measures (e.g. social distancing, crowd control, face masks) on the other hand. During the data collection phase of the current study, the meeting place was open for visitors all day. It was only at the time of writing that Villa Voortman was forced to close its doors again and go back to providing ‘through the window’ service due to a rising number of COVID-19 cases among staff members and visitors.

To obtain a wide diversity of perspectives on the place-making dynamics of the meeting place, a total of 14 participants (3 females, 11 males) were recruited from different groups of actors: visitors (n = 8), paid and unpaid staff members (n = 6). Staff members were purposefully recruited based on their function in the meeting place (e.g. psychologist, coordinator, mental health nurse, music therapist, volunteer, intern). The visitors of the meeting place were invited to take part in the study by the first author (CDR) during the weekly ‘visitors meeting’. Additionally, some (otherwise regular) visitors that had lost touch with the meeting place during the COVID-19 pandemic were purposefully recruited, to ensure the perspectives of ‘non-active’ visitors were also included.

### Data collection and analysis

Data were collected by means of in-depth interviews. Most interviews (n = 10) were conducted in a one-on-one format. However, some participants expressed their preference for a duo or group interview. Taking into account these preferences, and to increase service user involvement in the study, one duo interview and one group interview with seven participants were also included as data. During these conversations, participants were asked about the impact of the corona measures on the daily practice, principles and relational dynamics of the meeting place. All interviews took place at a location of the participant’s choice (e.g. Villa Voortman, participant’s house) or were conducted during a walking interview, and lasted between 16 and 71 min. An unforeseen added value of the group interview was that it encouraged service users to share their experiences and sparked a dynamic exchange of perspectives between service users and staff members, which positively impacted the depth and level of nuance in the results.

All interviews were audio-recorded, transcribed verbatim and analyzed by means of thematic analysis, using a phased approach [33]. In the initial analysis phase, the first author (CDR) familiarized with the data by reading the transcripts multiple times with a specific focus on the role and impact of place-making dynamics on the meeting place’s daily practice and service users’ recovery processes, keeping track of emerging themes and analytical insights. In the second round of data analysis, emerging themes and subthemes were clustered and all data were reanalyzed using this thematic structure. During this phase, emerging thematic insights were triangulated, nuanced and deepened by the day-to-day observations of coauthors DB and WH (who were employed at the meeting place at the time of data collection) regarding the meeting place’s atmosphere, daily practice and lockdown measures. In the third phase, a meeting was organized in which the first author presented preliminary insights to the coauthors and the thematic structure was extensively discussed. Based on the insights from this meeting, the thematic analysis was adjusted and finalized.

### Ethical considerations

This study was granted ethical approval by the Ethics Committee of the Ghent University Hospital (B670201628401). Written informed consents were obtained from all participants and data were processed in a pseudonymized manner.

## Results

By studying the place-making dynamics of the meeting place, it immediately becomes clear how rituals play a central role in shaping its daily practice. These rituals create a stable foundation and a safe harbor for the visitors of the meeting place, and become visible through the continuous (re)production of ‘hotspots’ in space and time. At the same time, place-making rituals are engaged in a continuous balancing act with the at times acute mental health and social needs of the visitors and the ‘survival rituals’ they bring into the meeting place. Another driving place-making force within the meeting place are the many creative and artistic processes, through which the meeting place opens up its pores to the outside world and visitors (re)create a language to engage in human encounters. In what follows, these dynamics will be discussed in detail.

### The (beating) heart of the matter: rituals of hospitality

One of the most characteristic rituals of the meeting place is the way both visitors and staff members greet each other very cordially, often accompanied by a kiss or a hug. This physical closeness, which is exceptional for a therapeutic setting engaging with this population, is a thread throughout many of the place-making rituals of Villa Voortman. Several participants pinpointed the essentiality of this ritual:

*“It is almost impossible to arrive at the villa and not greet everyone. If you don’t do that, it is somewhat incomplete. Something’s not right if you don’t do that. Not everyone kisses or hugs, but still… there’s something that needs to happen. And if that something doesn’t happen, something’s wrong. You can feel it immediately, if someone doesn’t greet you, then something’s… something’s not going well. Either that person is not feeling well, or… something’s wrong with the vibe…”* (staff member)

*“Yeah, since people don’t hold each other anymore and stuff, they will… You have less connection with each other. Otherwise, you come in, you give each other a hug, and you feel more connected.”* (visitor)

Another ritual that stands out is the daily process of cooking and eating lunch together. This ‘lunch ritual’ is an important act of shared territorialization in the meeting place, as visitors take ownership over the entire process, from deciding what to eat over cooking and eating together to doing the dishes afterwards. Due to the existing corona measures, this ritual was stripped of its essence, as visitors were no longer allowed to enter the kitchen, help in the cooking process or eat all together. Instead, cooked meals were provided by the nearby hospital. Whilst this made it possible for the meeting place to be open all day, a staff member explained how it also had a detrimental effect on the ownership visitors usually have over this ritual:

*“So the [cooking together] is no longer possible. (…) And the activity is gone, but also the choice. People can no longer choose. (…) They don’t even have the choice to say ‘hey mate, I’m not helping’. They don’t have the choice to decide what to eat. There is no choice of whether or not to go shopping. That really does have a big impact. (…) So all that foreplay for that meal, anyway, what precedes it… Sometimes fierce discussions, but it is interaction that we now miss.”* (staff member)

Place-making rituals are also marked in space and time and become visible in the continuous (re)creation of ‘hotspots’ in the meeting place. One of these hotspots is the sitting area, described by participants as *“our common living room”* or *“the heart of the villa”*, as it is a place buzzing with interactions and physical closeness. To comply with corona measures, the sofas of the sitting area have been removed, preventing too many visitors to sit together too closely. This is not just a loss of physical space, but also implies a fragmentation of the relational processes and physical proximity sparking in that hotspot:

*“I find it a shame that the living room is gone, because that’s where the day started. (…) All together, cozy in the sofa with a cup of coffee, chatting a bit, laughing a bit, fighting a bit, whatever. But that’s where the day started. The heart of the villa, that’s where it was. (…) But in that living room is where things happened. (…) Many conversations just don’t get started anymore, because the space is not there. (…) And you sit close to each other, because the living room doesn’t have enough capacity to all sit there. So often you have to sit buttock to buttock, like ‘man, move up!’.”* (staff member)

*“What I miss the most? Yeah, the sitting together and stuff. Yeah, we really had our corner here. And there [points to the dining area], it’s just not the same.”* (visitor)

Likewise, the fact that the kitchen became forbidden terrain for visitors due to corona measures is experienced as a big loss, as it prevents visitors from making a pot of coffee, clean up after spilling something in the sitting area, have informal conversations during washing up, leave a mess or help with cooking. Whilst these acts seem mundane at first sight, they turn the kitchen into a hotspot in which the horizontality between staff members and visitors is continuously rebalanced. One of the staff members, expressed his worries about the danger of fragmentation that the temporary ban on kitchen access might bring along:

*“What we see now is that we are actually splitting up a lot. We [staff members] are still allowed in the kitchen, the visitors are not allowed in the kitchen, still not. [During the first lockdown], we were allowed inside [the building], they had to stay out. (…) They are not allowed to make coffee themselves. (…) We are in danger of getting back to that… sort of hierarchical thing, or at least that separation, that’s the team and that’s the visitors. And that affects our structure.”* (staff member)

In addition to the small rituals (e.g. cooking, making coffee) and hotspots (e.g. the sitting area) that are continuously (re)performed and (re)created every day, multiple participants also pointed to the impact of bigger and less common rituals such as the annual Christmas party or certain artistic events (cf. infra), that leave important markings in time, strengthen the feeling of togetherness and have an expanding effect on the relational atmosphere in the meeting place, as one of the visitors explains:

*“Whenever we organize something big, there is always some kind of echo, a kind of afterglow, a kind of reverberation. (…) After something really good there is a more positive atmosphere in the villa for a while. And people are more cheerful and positive. Just like after the Christmas party.”* (visitor)

Although the corona measures were often experienced as barriers to performing place-making rituals and a cause for fragmentation of hotspots, they at times also gave rise to an intensification of these hotspots and a search for new ways to perform rituals. For example, during the first lockdown, the meeting place was only opened during limited opening hours to hand out meals through the window. At times, this single moment turned into a heated concentration of interactions and dynamics, characterized by heated encounters between visitors, a chaotic atmosphere and a more compelling demand for care and support. At other times, the temporary limited access to the meeting place made visitors actively search for other positive ways to create new hotspots in the surrounding area. One of the participants made a striking comparison in that respect:

*“[In the first lockdown], we became a kind of take-away restaurant, where people stuck around by the door. It created a different dynamic, which was fun sometimes, as long as the weather was nice. Maybe it was even more fun because you could see some of the old atmosphere coming back. Like visitors who manage to take a table outside and get installed in the park, and then you just think… Awesome.”* (staff member)

### Rituals as a counterweight: the entanglement of care and the encounter

Through the performance of place-making rituals, the meeting place is ongoingly (re)produced as a welcoming, trusted and literally ‘home-made’ environment. Whilst these rituals provide a sense of stability and familiarity for the visitors, they are also subject to and interacting with influences and interruptions both from within and outside the meeting place.

Because of the complexity of the visitors’ needs, it is not uncommon that they arrive at the meeting place in a state of crisis, caused by (an accumulation of) factors such as an acute psychotic episode, exhausting homelessness, intense substance use, social isolation and persistent confrontations with social exclusion. These factors are not characteristic to the meeting place, but they are inherent to the lives of the visitors through whom they find their way in. One of the visitors offered a striking example of the way homely rituals can have a cushioning effect that takes the edge off such crisis situations. Also, through his example, it becomes clear how the current decimation of hotspots such as the living room, due to the corona measures, obstructs these softening interactions:

*“So think about Jamie. Jamie doesn’t communicate with most people, and he is aggressive. But if there would be a living room, the chances would be much bigger that he… If it would be more homely, if we could cook together… Then there would be more opportunity to get in touch with him, to let him come down from his psychosis a bit. (…) The homeliness has a huge impact, you see? If you are raging, and then you enter the villa… Then poof. It is soft, it is warm, people are friendly. And that has a huge impact on the way you see things. And now, when you enter, it is like a visitor’s room in prison.”* (visitor)

Outside the meeting place, many of the visitors have developed strategies to cope with difficult living circumstances. Whilst these behaviors (e.g. substance use, small thefts, picking things out of bins) are considered unacceptable or destructive in society, they serve as important ‘survival rituals’ in the daily lives of the visitors. As such, these behaviors also find their way into the meeting place and endanger the safe climate that is continuously reproduced through place-making dynamics. However, there also is an advantage to these ‘survival rituals’ coming in, because that way, they can be questioned and counterbalanced in a non-intrusive manner within the meeting place.

*“Sometimes it is good when street life comes inside [the meeting place] a bit, so that some of the normality that we try to install here, like… So that you can hold up a mirror to them, like saying ‘guys, you don’t do that here. (…) You can’t rummage through the waste bins here’.”* (staff member)

The above examples show how the human encounter, that is continuously (re)produced in the meeting place, is an essential mediator in responding to the visitors’ needs. This entanglement is pre-eminently materialized in rituals that physically bring people closely together in the meeting place, such as sitting packed together in the living room, greeting each other with a kiss or a hug and smoking cigarettes together on the curb. In that sense, the necessary rules on social distancing to keep COVID-19 at bay are experienced as a big burden by all actors in the meeting place, which means that they are not always strictly adhered to. However, it would be too easy to interpret situations in which social distancing measures are breached as acts of carelessness or nonchalance. They rather bear witness to moments of extra carefulness in which the urge to meet visitors’ mental health needs takes the upper hand. One of the staff members explained how in certain crisis situations, the rule to avoid physical proximity simply becomes untenable, because providing that proximity is one of the most powerful ways to create an atmosphere of security and safety:

*“For example, when someone finds himself in a crisis, like last summer… When someone’s suddenly really psychotic and panicked… Yes, I held him then. Maybe I shouldn’t have, but at that moment, I had nothing else to offer but to, yeah… say like… I am here with you. And… Yes, that moment, you are almost… I couldn’t think of anything else than to offer safety, and I could only do that by providing it in a physical way.”* (staff member)

Through the illustrations described above, it becomes clear how place-making dynamics are engaged in a continuous balancing act with the at times challenging behavior and compelling needs that visitors express in the meeting place. Unsurprisingly, the COVID-19 pandemic caused a remarkable increase of crisis situations and an intensification of visitors’ requests for help with an array of social and personal problems. This rise was compounded with the fact that other social organizations (e.g. social council) and administrative instances (e.g. the bank) became much less accessible, turning the meeting place into ‘the final station’ for many visitors. As an initial reaction to this rise of support needs, staff were tempted to excessively focus on trying to fix individual visitors’ complex problems and to pay less attention to the more collective place-making dynamics in which the meeting place’s functions of being a safe, accepting and homely environment are continuously reproduced. However, the above examples show how exactly these dynamics play a pivotal role in dealing with these challenging situations. In that respect, one participant stressed the importance of prioritizing investing energy into these collective place-making rituals instead of endlessly trying to fulfil visitors’ needs:

*“I think we shouldn’t get lost in providing care. And that is a possibility, because yeah, that care is never sufficient, so it is up to you to decide how far you go. And you can go too far too, in that you lose the aspect of the encounter. And there are some people, who ask loads from us. Hours of talking, trying to get paperwork done, but it is never enough. Because there is a structural issue. Every time… It sounds like a strange thing to say, but that you feel like, it can’t be solved. (…) The lesson learned is that it is unsolvable, and that at a certain moment, you need to prioritize the encounter in what you do.”* (staff member)

Responding to the visitors’ complex needs thus requires a counterbalance that comes into being through the creation of a supportive and homely environment that facilitates human encounters (cf. supra). At the same time, the above citation shows how visitors’ needs can also put pressure on and even jeopardize collective place-making rituals. In that sense, the continuous interplay between visitors’ individual needs and collective place-making dynamics could be described as a relationship of resonance. Depending on different forces at play, they could either have a softening or an amplifying effect on each other.

### The art of place-making: creativity fueling place-making dynamics

The above findings show how the enabling dimensions of the meeting place are shaped and reshaped through place-making rituals that are engaged in a continuous balancing act with the complex needs of the visitors. Another driving force behind the production of the meeting place can be found in the many creative and artistic activities taking place. These take many shapes and sizes, such as making music, creating artwork (e.g. painting, sculpting), decorating the meeting place, gardening, cooking and dancing, to just name a few. Some of these activities are channeled through so-called ‘ateliers’, recurring workshops organized by the visitors themselves to inspire fellow visitors. However, many of these creative processes also find their way outside of these ateliers and continuously pop up in the meeting place, creating a kind of communal creative undercurrent that is contagious for other visitors. At the same time, because these creative processes are fueled in a bottom-up way by the unique talents and interests of the visitors, they can be considered deeply personal place-making dynamics through which visitors make the meeting place their own, and discover and (re)produce a language to engage in encounters with others. In that way, each visitor leaves their unique mark on the meeting place’s character.

*“You can do your own thing here, they’re open to anything. They even want to provide materials for you. You can do your thing. If you want to tinker, tinker. If you want to draw, just draw. If you don’t want to do anything, do nothing. (…) This morning, there was a bike here, they were going to throw it away. I took all the locks off, fixed it, pumped up the tires and stuff.”* (visitor)

One afternoon per month, these personal place-making traces are brought together in a so-called ‘Open Gate’ event. During these events, the meeting place opens its doors to external guests, for who the visitors perform live music and spoken word over homemade cakes and coffee. Time and again, in the run-up to this monthly happening, an energetic vibe is sparked in which visitors are busy rehearsing their performances and the ‘Open Gate’ becomes the talk of the moment. In that sense, the ‘Open Gate’ event can be seen as a kind of collective ritual through which feelings of togetherness are strengthened and spontaneous outbursts of creativity emerge:

*“When there is an Open Gate event, then people are rehearsing, they are thinking about what they’ll perform or… Yeah, I find those afternoons, there’s always a really cool vibe of like… Soon our doors will open and people will come in. And part of it is prepared and I like that a lot, but there are also a lot of things happening spontaneously in the moment… Like people who say no, I won’t perform, at first but are then triggered in the moment to do something. (…) In those moments I feel so proud of the villa. I don’t even do anything, I just make coffee and cake or so.”* (staff member)

The ‘Open Gate’ events also illustrate how creative dynamics open up pores in the meeting place through which visitors interact with the outside world. It is through these on-going porous interactions that visitors feel empowered, (re)build their identity as artists and people with talents, and (re)claim a place as visible citizens in society. It needs no explanation that since the outbreak of the COVID-19 pandemic, organizing these ‘Open Gate’ events and inviting guests into the meeting place temporarily became impossible. However, after a while, the idea arose to turn them into ‘Closed Gate’ events, in which visitors’ performances are livestreamed via social media. Despite the fact that visitors thus had to perform behind closed doors, it became apparent how these ‘Closed Gate’ events did restore some of the familiar buzz that swells in the build-up to that monthly afternoon. Additionally, despite the virtual audience, visitors expressed that they still felt seen by the outside world, perhaps even more so than before, as social media have a wider reach.

Throughout the years, these internal creative place-making dynamics have increasingly also found their way on another level, as the meeting place has been establishing itself in the professional cultural circuit by creating and performing large-scale theatre and music productions, supported by professional directors and producers. The composing of these productions, the long preceding rehearsal period, the thrill of performing in sold-out venues and the long-lasting afterglow of these theatre adventures have an enormous impact on the place-making dynamics within the meeting place. Moreover, through these large-scale productions, the meeting place’s pores to the outside world get spread open wide. In that sense, the fact that these productions are all temporarily suspended is experienced as a big loss, not only for the sake of these productions in themselves but especially for the place-making waves that it produces in the meeting place. One of the staff members expressed his concern in the following way:

*“We have lost the core business of the villa. Probably no Indian Summer event, no performances,… All these occasions where we came out, where we showed a different side of our visitors, which is very important… We’ve lost it now, because of corona.”* (staff member)

A side effect of these professional productions is that the meeting place increasingly acquired the image of being an alternative artistic collective, rather than a day-care center for persons with complex mental health needs. Whilst some staff members and visitors strongly identify with this image of the meeting place as an artistic hub, it also has an adverse effect for others. For example, according to one of the visitors, the day-to-day place-making rituals risk becoming somewhat paralyzed during times in which the meeting place is engaged in rehearsals for big artistic projects (e.g. ‘Avanti!’, a theatre production), as all energy is channeled towards these projects. In contrast, the fact that large-scale artistic productions are currently impossible due to the COVID-19 pandemic implies that the space to perform these everyday place-making rituals remains intact:

*“I used to find the Villa much less accessible during ‘Avanti!’ [theater production] than during the corona crisis actually. Because in those days, there was nothing left in the villa, all that mattered was ‘Avanti!’. (…) There was nothing left of the usual things. Everything revolved around the theatre and nothing else.”* (visitor)

Through this example, it becomes clear how the meeting place’s artistic productions create an ambiguous dynamic. On the one hand, these productions spark a bustling vibe amongst the visitors, give purpose to the creative juices flowing and hold the potential to cause long-lasting ripples of positivity in the meeting place’s atmosphere (cf. supra). On the other hand, they run the risk of disturbing or subduing the more everyday rituals, resulting in visitors feeling unsettled and struggling to feel at home in the meeting place. In that respect, precisely because large-scale artistic productions are currently not possible, the COVID-19 pandemic could be seized as an opportunity to bring creative dynamics in the meeting place back to their essence, according to some participants. For example, one participant described how artistic talents and processes should not always take place in function of a stage performance or a professional production, but should first and foremost be valued as dynamics that play a pivotal role in shaping the meeting place and enabling visitors’ recovery processes:

*“Alright, you want to meet people via creativity, which makes up a large part of the villa. But it shouldn’t always be in function of a stage. And that’s a thin line we walk, sometimes. (…) [I think the corona crisis] provides people with a bit more peace and you can just play, just purely because of the music. And purely because you want to have fun, and use the music for what it’s intended. And not in function of [a performance].”* (staff member)

Obstacles caused by the corona crisis (e.g. not being allowed to share music instruments, not being able to perform for an external audience, social distancing rules) did not wipe out these creative place-making dynamics. Proof of this can be found in the many small creative projects that sprouted in and around the meeting place during the height of the pandemic, in different shapes and sizes. For example, some of the visitors spontaneously organized a small acoustic jam session on the meeting place’s motorized boat, cruising through the city center and enlightening casual passers-by with their songs. Other visitors built a doghouse for the dog of one of their fellow visitors as a welcoming attempt to get her to visit the meeting place more regularly. An infectious trend arose amongst the visitors to decorate the walls and furniture of the meeting place with their own paintings, drawings and writings. The idea arose to install a homemade totem pole as a landmark outside the meeting place. Visitors kept performing music and spoken word, albeit behind closed doors, during the monthly ‘Closed Gate’ events and at many other stolen moments. These are just a handful of examples that show how small-scale acts of creativity are indispensable place-making dynamics that fuel the (re)production of the meeting place’s unique identity. As stated above, it is through the continuous (re-)enactment of such creative processes that visitors find a language to engage in human encounters and the meeting place is turned into a porous entity interacting with the outside world. In that way, the creative and artistic products that the meeting place brings forth, both small (e.g. poems, drawings, music) and big (e.g. theatre productions, books, music performances), could be seen as materializations of place-making rituals and dynamics, leaving tangible traces like unique fingerprints in space and time.

## Discussion

Through scrutinizing the place-making dynamics of one low-threshold meeting place, the aim of the current study was to open up the black box of recovery processes in persons with complex mental health needs. In doing so, the importance of enabling environments as complex sites of interpersonal relations came into focus. Studying the place-making dynamics of Villa Voortman, the focus was explicitly set on the reciprocal dynamics between service users, the meeting place and the broader societal context, rather than the one-way impact of the meeting place’s practice on the recovery processes of its users [24]. Through studying these place-making dynamics during the height of the COVID-19 pandemic, they became extra visible, as they were extra articulated, took on a new form or became visible marked by the contours of their absence. This allowed us to discern and better understand the characteristics of these enabling environments and the interpersonal elements that play an essential role in the recovery processes of persons with complex mental health needs. Firstly, recovery processes thrive in environments characterized by a radical *hospitality*, an openness to the otherness of the other free from judgement, societal norms and expectations. Secondly, and related to the former, supportive interpersonal relationships are rooted in *horizontal* relational dynamics, rather than top-down treatment relationships and power imbalances. Thirdly, recovery processes are sparked and fueled through the continuous *reciprocity* of ritualistic interactions dynamically bringing together and re-enacting the material, social and affective dimensions of recovery-enabling environments. Finally, the current study showed the importance of the *porosity* of enabling environments allowing place-making rituals to cause ripples and waves that reach beyond these environments and impact service users’ experiences of citizenship, identity and belonging. In what follows, these aspects will be further discussed.

The results showed how the daily practice of low-threshold meeting places such as the one under study is shaped through the continuous performance of place-making rituals. These rituals are typical of the meeting place and differ from other dynamics that visitors are usually confronted with in their daily lives. For example, they differ from the ‘survival rituals’ (e.g. substance use) that visitors are compelled to perform in order to cope with vulnerable living circumstances (e.g. street life, social isolation). Likewise, they stand out from dominant societal practices and traditions that persons with complex mental health needs are often excluded from, incited by experiences of stigma and marginalization [34]. In addition, throughout their long and often unsuccessful treatment trajectories, persons with complex mental health needs have in many cases been confronted with hospital logics, that are colored by top-down regulations and power relationships [35]. These experiences are in contrast to the place-making dynamics shaping the meeting place. Although different actors (visitors, staff members, volunteers) bring different perspectives (e.g. professional ethos, interests, lived experience) into the meeting place, these differences do not lead to dominant power dynamics and top-down ritualistic practices. Instead, it is precisely through the dynamic encounters between these different perspectives and actors that new peer group rituals are created and reproduced in a bottom-up way. In that respect, the results of this study pointed to the essentiality of everyday rituals such as greeting each other cordially with a kiss and a hug, eating and cooking together, and the on-going creation of hotspots.

These seemingly mundane or domestic rituals bring the meeting place to life as a territory of recovery [24, 36], which atmospherically can be situated somewhat between a place of refuge and a sanctuary. Within this dynamic space-in-between, one of the core values that is continuously being reproduced is hospitality, an active welcoming of the other, be it in different guises. First, through the performance of these rituals, an enveloped space of safety, equality and inclusion is created in which people’s particularities appear, hence, are not a priori problematized. In other words, in these seemingly trivial events lies an active invitation towards those aspects of one’s reality that are predominantly rejected by society. For example, in the meeting place, one’s substance use or psychosis is not problematized in itself or seized as a reason for exclusion, but is seen as an inherent part of one’s reality. This resonates well with ideas from Derrida’s essay on hospitality, in which he suggests that hospitality implies a fundamental right to protection and shelter for those who are vulnerable and on the run from places where life is no longer safe [37]. Visitors of a meeting place like Villa Voortman are not refugees in the literal sense of the word. Yet, they struggle with personal issues like debilitating psychotic experiences, encounter a health care and welfare system that often does not respond to their needs, and are haunted by societal demands they cannot comply with. Hospitality can counter the resulting estrangement and again help to connect and feel recognized for the individual that you are. Derrida indicates that hospitality starts with a non-judgmental openness for the otherness of the other, and builds on mutual generosity. Language, he says, is hospitality, because in the act of inviting, welcoming, giving shelter and mere talking to one another, hospitality is performed. Such openness, generosity, and culture of speaking openly is crucial to the place-making dynamics observed in this study. Visitors are welcomed and not measured against performance standards or criteria of normality. COVID-19 confinement measures challenged practices of hospitality, but made clear that seemingly simple rituals like having coffee and hanging out at hotspots are absolutely crucial.

Hospitality is not only reproduced within the walls of the meeting place, but also radiates and expands to the outside world. In that respect, the results showed how the many creative processes and artistic activities turn the meeting place into a porous entity that is in a continuous dynamic interaction with its surrounding context [38], which has a twofold effect. On the one hand, from a Derridarian perspective, place-making practices in Villa Voortman imply fluidity in the guest–host role [39]. The permeability of the meeting place instills a dialectic in which visitors are enabled to take on the role of hosts that actively welcome ‘outsiders’ into the meeting place. Through these external visitors, societal rituals and practices are pulled into the meeting place, allowing them to interact with the meeting place’s own particular rituals. For Derrida, it is precisely this openness and acceptance of the ‘other’, albeit on the terms of the host, that provides the host with the opportunity of new experiences [39]. In other words, because of this radical and welcoming openness, visitors are encouraged and invited to rebuild a sense of sense away from spoiled and problem-focused identities (e.g. mentally ill, addicted, homeless) and to establish a unique language to engage in dialogue with others [32]. On the other hand, through activities such as the ‘Open/Closed Gate’ events and theatre productions, the meeting place appeals to and evokes the hospitality of the neighborhood and society, thus actively claiming visible citizenship of its service users [32]. This shows how inclusive citizenship, that is often put forward as a pivotal dimension of mental health recovery [40, 41], is not just a static recovery-oriented objective. Instead, it is an on-going dynamic practice that is fueled and reproduced through the relational dynamics of hospitality both within and between the meeting place and the outer world.

The results showed how the preventive corona measures at times profoundly obstructed these essential rituals of hospitality, as everyday practices (e.g. eating and cooking together) were temporarily suspended, the daily practice of the meeting place was forced to fold back on itself, and the meeting place’s degree of permeability was reduced to a minimum. Despite its detrimental impact on the daily practice of Villa Voortman, the COVID-19 pandemic did provide us with a unique research window to gain further understanding of the dimensions of recovery-enabling environments and the ways recovery processes and place are interlinked. First, the consequences of the COVID-19 confinement measures on the place-making dynamics of Villa Voortman confirmed how enabling environments should be seen as dynamic concentrations or *“site[s] of complex relations”* [42] (p. 415) rather than static entities, in which recovery resources come to life through the fluctuating encounters between an individual and their surrounding world [23]. In that sense, the enabling properties of places such as the meeting place should be seen as experiences that are generated precisely in the unique and dynamic convergence of the different material (e.g. a cooked meal), social (e.g. the proximity of other visitors) and affective (e.g. feeling safe) resources of that place [21]. Second, the results of the current study tally well with the framework of Doroud, Fossy and Fortune [20] in which place is put forward as an essential facilitator of recovery through mechanisms of being, doing, becoming and belonging. Indeed, the meeting place provides visitors with a sense of homeliness and security (*being*), enables creative processes and artistic activities (*doing*) through which visitors (re)connect with their identity and find a language to engage in dialogue (*becoming*), and creates positive ties to the community (*belonging*) [20]. From that perspective, it is fair to assert that the meeting place facilitates and supports the recovery processes of its visitors, persons with complex mental health needs. However, the results of the current study revealed that this should be seen as a reciprocal dynamic rather than a one-way effect, as it is only through the continuous re-enactment of these ritualistic interactions that the material, social and affective recovery resources of the meeting place are produced over and over again [21].

In parallel, the results of the study also echo the five subprocesses of personal recovery as stipulated by Leamy and colleagues (2011) in the CHIME framework. However, by studying recovery in persons with complex mental health needs through a relational geographical lens, an important nuance related to this popular recovery conceptualization came to the fore, that can be illustrated by reflecting on the many creative and artistic processes and activities taking place in the meeting place. Regardless of how small or large-scale these artistic processes are, they primarily spark as personal and collective place-making dynamics through which visitors (and other actors) make the meeting place their own. In other words, they are not intentionally brought forth with the aim to strengthen Connectedness, generate Hope, build Identities, give Meaning or engender Empowerment [13]. Instead, these creative and artistic processes are the ground from which these recovery dimensions dynamically emerge, as side effects of the place-making dynamics in which they are ingrained. In line with this, Duff (2016) asserts that *“recovery is made of these moments, and their social, spatial affective and material durations whereby novel capacities emerge and coalesce”* (43, p. 71). In that sense, insights from the current study can be seen as a wake up call as to not overly psychologize these recovery dimensions as intrapersonal characteristics, linear processes of growth or static (outcome) indicators of treatment, but to see them as continuously evolving interaction processes [43]. From that perspective, it becomes impossible to separate the individual in recovery from the context in which these on-going interactions are (re)produced over and over again [15].

Finally, the results of the current study are indicative of the way support and treatment for persons with complex mental health needs can be organized in a more adequate way. Existing research has shown that specialized treatment modalities often remain characterized by a persistent focus on symptom stability, crisis management and a poor link between complex mental health needs and the context in which they come about [2, 8]. As persons with complex mental health needs have difficulties finding connection with such an overly clinical and outcome-oriented logic, their image as a ‘hard-to-reach’ population unintendedly risks being reinforced [44]. At the same time, previous research has stressed the importance of peer support, mutual aid and community involvement to meet the support needs of persons with complex mental health needs [45–47]. Building on these insights, this study showed how alternative low-threshold treatment approaches such as Villa Voortman, that radically put the human encounter and relational connection at the center of their daily practice, can have a greatly empowering effect on the lives and recovery processes of persons with complex mental health needs [32].

## Conclusions

The meeting place under scrutiny in this study revealed a radically different approach to support for the complex and heterogeneous population of persons with complex mental health needs. Through the place-making dynamics at play, hospitality is reproduced time and again, curating an inclusive space in which the human encounter, rather than a primary focus on individual needs, is placed at the heart of its daily practice. Within such a space, recovery processes such as Connectedness, Hope, Identity, Meaning in life and Empowerment (cf. the CHIME framework, [13]) come to life through dynamic interactions or *“lived, affective practice[s] of becoming well”*, as Duff (2016, p. 68) describes it [43]. In that sense, this study made clear that in our attempts to understand and support recovery in persons with complex mental health needs, we have been blinded for too long by an excessive focus on the individual in recovery as primary unit of analysis [48]. In conclusion, further opening up the ‘black box’ of recovery in persons with complex mental health needs in future research requires a shift in our analytic gaze towards recovery as a relational assemblage, to say it in a Deleuzian way, rather than a purely intrapersonal process [25]. By focusing on the lived experiences of the meeting place’s service users and staff members, we made the explicit methodological choice to study the role of place-making dynamics from an insider perspective. Future research is necessary to deepen our understanding of these dynamics, by further contextualizing them socially and culturally, and by studying the impact of macro-level societal developments on these relational assemblages.

## Data Availability

The datasets used and analyzed during the current study are available from the corresponding author on reasonable request.
